# Small Contaminations on Broiler Carcasses Are More a Quality Matter than a Food Safety Issue

**DOI:** 10.3390/foods12030522

**Published:** 2023-01-24

**Authors:** Kacper Libera, Len Lipman, Boyd R. Berends

**Affiliations:** Institute for Risk Assessment Sciences (IRAS), Utrecht University, 3508 TD Utrecht, The Netherlands

**Keywords:** food safety, poultry, slaughter, carcass, contamination, Monte Carlo simulation, process hygiene criteria

## Abstract

Depending on the interpretation of the European Union (EU) regulations, even marginally visibly contaminated poultry carcasses could be rejected for human consumption due to food safety concerns. However, it is not clear if small contaminations actually increase the already present bacterial load of carcasses to such an extent that the risk for the consumers is seriously elevated. Therefore, the additional contribution to the total microbial load on carcasses by a small but still visible contamination with feces, grains from the crop, and drops of bile and grease from the slaughter line was determined using a Monte Carlo simulation. The bacterial counts (total aerobic plate count, *Enterobacteriaceae*, *Escherichia coli*, and *Campylobacter* spp.) were obtained from the literature and used as input for the Monte Carlo model with 50,000 iterations for each simulation. The Monte Carlo simulation revealed that the presence of minute spots of feces, bile, crop content, and slaughter line grease do not lead to a substantial increase of the already existing biological hazards present on the carcasses and should thus be considered a matter of quality rather than food safety.

## 1. Introduction

The muscles and internal organs of healthy slaughter animals are normally sterile, but during slaughtering, both the carcasses and internal organs invariably become contaminated with bacteria. Historically, the main factors affecting the final bacterial load of carcasses and consequently cuts of meat are driven by the cleanliness of the slaughterhouse environment and the skills of the slaughterhouse workers. However, increased mechanization has considerably reduced the human role in controlling the bacteriological quality and safety of meat. Today, the level of poultry carcass contamination is predominantly determined by the performance of the slaughterhouse machinery and the bacteriological status of the animals pre-slaughter. For example, not maintaining a constant high temperature (e.g., due to thermostat malfunction) in scalding machines increases the chances of carcass bacterial contamination by almost five times [1], and with respect to the bacteriological status of the animals pre-slaughter, *Campylobacter* spp.-positive flocks (positive caeca contents) are approximately four times more likely to cause *Campylobacter* spp. contamination of the carcasses at the end of the slaughter line compared to *Campylobacter* spp.-negative flocks [2]. Therefore, in a modern poultry slaughter line, the bacteriological safety and quality of the carcasses at the end of the line are ultimately determined by the number of bacteria present on and in the live animals as they arrive at the slaughterhouse in combination with the effectiveness and adjustment of the defeathering and evisceration equipment and that of the carcass washers [3,4,5].

Chicken carcass contamination continues to be a major food safety concern because broiler meat remains an important source of human campylobacteriosis. The latest data in the EU show that there were 120,964 confirmed cases of campylobacteriosis [6], and it is estimated that 20 to 30% of infections could be attributed to the handling, preparation, and consumption of broiler meat [7]. However, due to the self-limiting nature of this disease, the real prevalence is far higher. To design optimal interventions, it is crucial to understand how carcasses can become contaminated and which factors contribute to the contamination. Pacholewicz et al. [8] demonstrated that bacterial concentrations in the intestines of broilers are an important explanatory variable of carcass contamination because these were associated with fluctuations in *Campylobacter* spp. and *Escherichia coli* concentrations at various processing steps in the slaughter line. This is in accordance with a study performed by Tang et al., [9] who reported the highest prevalence of *Campylobacter* spp. contamination at the evisceration step due to the exposure of intestinal contents. In this study, the *Campylobacter*-positive carcass rate decreased from 53.4% during evisceration to 14.75% after cooling, which suggests that the cooling step is crucial for eliminating *Campylobacter* spp. on chicken carcasses. Furthermore, earlier research by Pacholewicz et al. [10] demonstrated that the changes in numbers of *Escherichia coli* and *Campylobacter* spp. on chicken carcasses during the various processing steps in a slaughter line are of a similar nature. However, a direct relationship between total bacterial load on chicken carcasses at the end of the slaughter line and the number of poultry-related cases of human disease have never been established, with the possible exception of a few risk assessment models for *Campylobacter* spp., such as the one described by Nauta et al. [11]. From the stable-to-table model of Nauta et al. [11] in particular, it was inferred that, in the slaughterhouse, compliance to a maximum threshold of about 1000 CFU/g of fresh chicken meat probably would halve the number of associated human cases of campylobacteriosis in the EU [7].

A strategy to reduce risk for consumers is to decrease the counts of *Campylobacter* spp. in the intestines of live birds with a range of control options available, including vaccinations, feed additives, or phage therapy. By lowering the concentration in the intestinal content, these control options aim at reducing *Campylobacter* spp. contamination during broiler processing and thus lead to lower concentrations on the broiler meat. A recent model suggests that a relative risk reduction (39%) could be obtained through a 2 log_10_ reduction in caecal concentration (9 log_10_CFU/g to 7 log_10_CFU/g) [12]. However, it is important to note that the association between concentrations found in caeca and skin largely depend on the variation in hygiene practices between slaughterhouses and regions; consequently, the scale of potential risk-reducing effects may also vary greatly [12].

In almost entirely mechanised processes, the biological variation and physical condition of the animals are the most important factors with regard to the occurrence of slaughter defects, i.e., damaged intestines and/or gall bladders [4,13,14]. However, quantitative assessments of fecal contamination that are the result of this practice are limited. In an article from 1997, Russell and Walker [13] reported that the American inspection services found 0.8 to 5% fecally contaminated carcasses just before cooling. The study of Russell and Walker also demonstrated that, just after evisceration, 4–6% of the broiler carcasses showed evidence of fecal leakage on the inside and 5.2–8.4% on the outside [14]. Brizio et al. [15] investigated different types of carcass contamination and reported that 6% of carcasses were found to have fecal contamination, 1.45% of the carcasses were contaminated with bile, while 1.90% were contaminated with gastric content. In total, 9.35% out of 51,500 examined broiler carcasses were contaminated. Another field study found that, at the end of the slaughter line, just before cooling, 2–5% of the broiler carcasses were fecally contaminated [16]. It is important to highlight that there are significant differences in the prevalence of visibly contaminated carcasses between slaughterhouses representing different levels of compliance with food safety procedures [17].

The total bacterial load of chicken carcasses is often considerable, regardless of the presence of any visible contamination. Cibin et al. [18] reported, in an EU study, that carcasses visibly uncontaminated with feces and sampled just after evisceration showed *E. coli* loads (log_10_ CFU/g) that ranged from 1.30 to 7.38 and that visibly fecally contaminated carcasses showed loads from 2.40 to 7.04, respectively. Visibly uncontaminated carcasses sampled just after cooling showed *E. coli* loads that ranged from 1.00 to 6.95, whereas in visibly fecally contaminated carcasses, counts ranged from 2.65 to 5.28, respectively. With regard to the *Enterobacteriaceae*, after evisceration, the visibly clean carcasses had loads that ranged from 1.48 to 7.45, whilst counts on visibly fecally contaminated carcasses ranged from 2.45 to 7.26, respectively. After cooling, the loads with *Enterobacteriaceae* ranged from 1.00 to 7.08 for visibly clean carcasses and from 3.54 to 5.18 for fecally contaminated carcasses, respectively. Research by Jimenez et al. [19,20] reported comparable figures from Argentinean poultry slaughterhouses. In addition, they also demonstrated that there were no significant differences in numbers of *Enterobacteriaceae*, coliforms, and *Escherichia coli* per gram or cm^2^ between visibly contaminated and uncontaminated carcasses. In contrast, however, *Campylobacter jejuni* and *Campylobacter coli* were detected in 58.8% and 11.6% (respectively) of broiler carcasses with visible fecal contamination, as compared to 17.6% and 9.8% in carcasses without visible fecal contamination [21]. However, the counts of *Campylobacter* spp. did not significantly differ between carcasses with and without contamination. At retail level, broiler carcasses are also characterized by an abundant microbiome, including pathogens as reported by Yu et al. [22], who found that 100% of organic carcasses were *Campylobacter*-positive compared to 8.33% in conventionally reared carcasses. Furthermore, 5 % of conventionally reared carcasses were contaminated with *Salmonella* spp., while the other most abundantly present bacteria included *Pseudomonas, Serratia* spp., and *E. coli*.

In 2022, the Association of Dutch Poultry Processing Industries (NEPLUVI) requested the Division of Veterinary Public Health of the Institute for Risk Assessment Sciences (IRAS) to estimate a) the total bacterial load of an ‘average’, visibly clean chicken carcass at the end of the slaughter line, b) to estimate what extent a small contamination would add to this ‘average’ total bacterial load, and c) to determine whether or not this would mean a substantial increase of any food risk already present that would make that carcass unfit for human consumption. Therefore, the aim of this study was to investigate the bacterial load on carcasses with different types of small but still visible contaminations with feces, crop content, and bile and grease from the line and compare these carcasses to those without any visible contamination with the use of a Monte Carlo simulation. We hypothesized that there are no significant differences in bacterial loads between carcasses with a small contamination and those with no visible contamination.

## 2. Materials and Methods

### 2.1. Data Used for Input in the Calculations

Bacterial counts (mean values of bacterial log_10_ counts ± standard deviation) used for the calculations were collected from peer-reviewed journals. The main selection criteria of articles included study design, performed laboratory analysis, sample size, year of publication, and parameters of the journal quality and impact. We aimed that the data from chosen articles should be representative and correspond as much as possible with a contemporary slaughterhouse environment.

### 2.2. Monte Carlo Simulation

A Monte Carlo simulation is a method used to predict the outcomes of events derived from multiple variations in their input [23]. It leads to insight into how ordinary or extraordinary certain final outcomes of these calculations are. The actual Monte Carlo simulation was performed using @Risk 8.0, which was part of the software package ‘Decision Tools Suite’ (Pallisade Corporation, 2020) and can be used as an add on to an Excel spreadsheet [24]. This method has already been successfully implemented to detect *Campylobacter* spp. presence and concentration using different chicken carcass samples [25].

In a Monte Carlo simulation, the variables that determine the outcome are repeatedly drawn from a range of values that follow a user-defined probability distribution [23,24]. @Risk was set to perform 50,000 iterations for each simulation.

**Figure 1:**
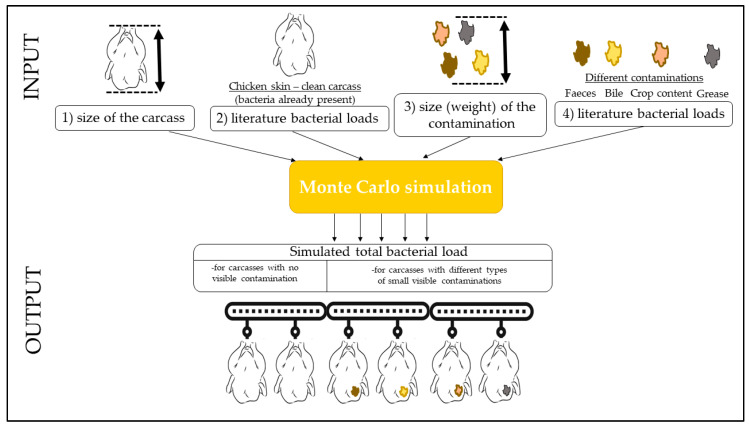
Data used for input in the Monte Carlo simulation.

The variables that were given a @Risk function were: (1) the total surface (weight) of the carcasses, (2) the number of bacteria per square centimeter (gram) already present on the skin surface of a clean carcass, (3) the total weight of a contamination, and (4) the total number of bacteria present per gram of contamination. A graphical explanation of the model design is given in Figure 1, and an example spreadsheet (Spreadsheet S1) model was uploaded in the Supplementary Materials.

The weights of the carcasses and the numbers of bacteria present on the carcasses and in the contaminations were processed with the @Risk function ‘normal distribution’ using values of mean and standard deviation. In order to avoid obtaining unrealistic results (e.g., lower than 0) of the total bacterial load prediction, we set the maximum and minimum value for the distribution of bacterial counts. For every bacterial count distribution, the minimum value was set to ‘0’, while the maximum number of bacteria varied between materials and was set at 10^9^ CFU/g for skin, 10^10^ for feces, 10^9^ for crop content, 10^8^ for bile, and 10^4^ for grease. A similar procedure was followed by Nauta et al. [26]. The weights of the contaminations were processed with the @Risk function ‘uniform distribution’ using minimum and maximum values (see Table 1) because we had no knowledge about the real frequency distribution of the weights of small contaminations [23].

To determine the total load with bacteria on a ‘typical’ broiler carcass, a calculation was conducted with the aid of results from the study by Elfadil et al. [27]. From their study, it can be inferred that approximately one gram of body weight equals circa 1cm^2^ surface. The spreadsheet model used an ‘average’ bird weight of 1600 g (i.e., 1600 cm^2^), since this corresponded to the weight of the smaller animals both slaughterhouses confirmed to regularly process, and it is to be expected that a contamination has the biggest impact on a relatively small carcass. The total carcass load was then calculated by multiplying the bacterial counts per gram of the chicken skin with the total surface of the carcass.

**Figure 2:**
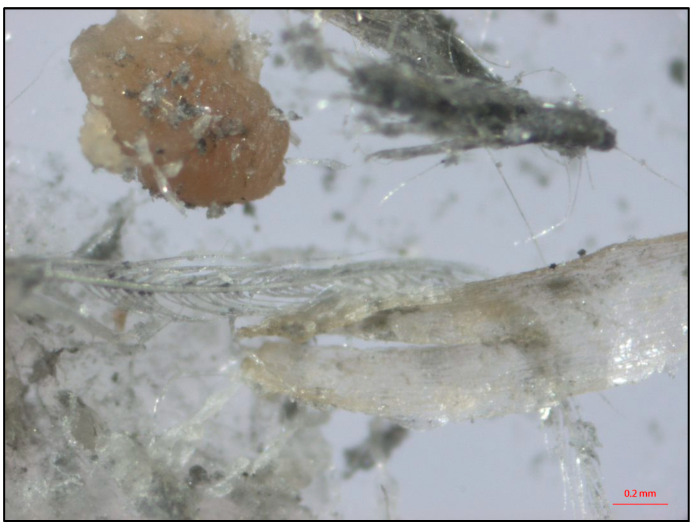
The microscopic view of the grease.

All small contamination sizes were identified and described using a standardized number of grains or droplets (crop content and bile, respectively) or circle-shaped spots (feces and grease). In combination with the specific weights of the materials involved, the mass of a contamination could then be calculated. For the crop content and the bile, we used the generally acknowledged international standards of 0.065 g for a grain and 0.05 mL for a droplet. The specific weight in grams of the feces and bile fluid was estimated using Cussler et.al. [28] and Van der Meer [29]. When microscopically examined, the slaughter line grease turned out to be a mixture of chicken skin and feather material, minute metallic particles from the line, and the original food grade lubricant (see Figure 2). Therefore, we assumed that the specific weight would be in between that of the weight of feces, bile, and crop content.

The variation in carcass weights was approximated based upon the average weight of batches of animals sent to slaughter having a standard deviation of 5%, and that in a batch, the lightest animals weigh, on average, minus three times the standard deviation and that the heaviest animals weigh the average plus three times the standard deviation [24]. In this case, the ‘average’ weight was set at 1600 g, the minimum weight at 1350 g, and the maximum weight at 1850 g. The weights of the different contaminations that were used as model inputs are listed in Table 1.

## 3. Results

The microbial literature data that were used as the input for the Monte Carlo simulation can be found in Table 2. To the best of our knowledge, there are no bacteriological data available for grease; thus, we used our own (not published) data. Similarly, there are no studies describing bacterial counts in the bile after it has leaked from the gall bladder onto the machinery and/or the digestive tract before it drips onto the carcass during the evisceration. Therefore, we used bacterial counts from liver samples, assuming that the bacteriological loads of the liver correspond with bacteria potentially present in the bile after it has leaked from the gall bladder onto the machinery and the gut.

The simulated total bacterial loads on the broiler carcasses (mean 1600 g) with or without small visible contamination are given in Table 3.

The probability that a small visible contamination results in at least a 0.5 (log_10_CFU) increase in the total bacterial load of the average chicken carcass is given in Table 4. The value of ±0.5 log_10_CFU is considered as the precision of classical microbiological methods [41]. From the practical point of view, differences below this value cannot be identified with classical microbiological culturing methods.

It is important to note that the difference of 0.5 log_10_CFU is roughly equivalent to a three-fold increase in the total bacterial load (calculated based on CFUx10^x^). The probability that small contaminations result in at least a three-fold increase in the total bacterial load of the average chicken carcass is given in Table 5.

It is also important to determine what the contribution is of small contaminations to the total bacterial load compared to the already existing bacterial loads on the carcass. Therefore, the percentual contribution of a small contamination to the final total bacterial load on the carcass is given in Table 6. In the majority of the simulations, it was below 1%.

**Figure 3:**
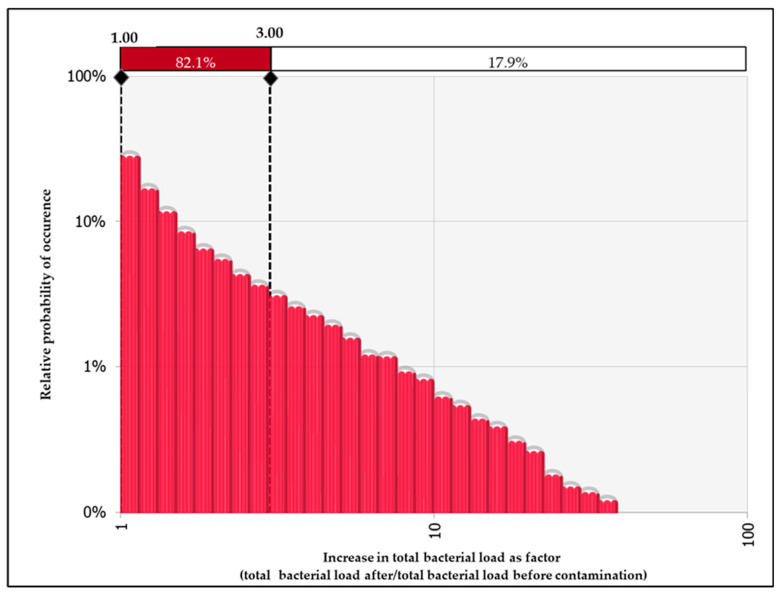
Relative probability of occurrence of different increases in total bacterial load expressed as a factor in the case of *E. coli* counts and contamination with feces. The red horizontal bar indicates the probability of increase less than three-fold (1.00–3.00), while the white horizontal bar indicates an increase more than three-fold.

The highest probability that the bacterial count increase is higher than three-fold (17.9%) was obtained in the case of *E. coli* of a carcass contaminated with feces. This relationship is visualized in Figure 3.

## 4. Discussion

### 4.1. Bacterial Counts Used for Calculations

As expected, feces contained the most bacteria, and they far exceeded the counts on chicken skins. It is not surprising that, in the literature, there is a lack of data regarding microbial counts in the bile. Normally, bile fluids should contain zero to very few bacteria, since, otherwise, the animals would develop cholecystitis and become clinically ill and unfit for slaughter. It is challenging to either investigate or simulate bile bacterial counts found after the machinery has damaged the gall bladder. Specifically, it is particularly difficult to determine the bacterial counts present in bile itself before it reaches carcasses because the bile is usually mixed with gut content before it contaminates the carcasses. We assumed that microbial counts from chicken livers would approximate that of bile that contaminated the carcass via the machinery and the viscera. Crop content bacterial counts resembled an intermediate level between that of the skin and of the feces contents. The lowest counts of bacteria were observed in grease, since this material is mainly composed of lipids (almost no water), consequently creating a hostile environment for bacterial growth.

### 4.2. Monte Carlo Simulation

The results of the Monte Carlo Simulation after 50,000 iterations are summarized in Table 3. The high number of iterations ensures that the simulation included almost all of the possible combinations of carcass weight (surface), clean carcass bacterial numbers, and weights of the small contaminations with corresponding bacterial counts of the small contaminations. For modern computers, a simulation with 50,000 iterations is not a challenge and lasts for approximately one minute. As expected, the highest differences between bacterial loads in contaminated and non-contaminated carcasses were observed in the case of fecal contamination. In other cases, if the differences existed, they occurred at the second to the fourth decimal place of bacterial counts.

We also determined the probability that the increase in the total bacterial load would exceed the precision limit of classical microbiology methods (0.5 log_10_CFU). This value is important because increases below this number cannot be identified by classical microbiological methods and can thus be considered insignificant. Similarly, the highest values were observed in carcasses contaminated with feces, in particular in *E. coli* counts (Table 4 and Table 5). For example, there was a probability of 16.7% that, after a small contamination with feces, *E. coli* counts would increase by 0.5 log_10_ (Table 4). In other words, roughly 1/6 of the carcasses with small visible contamination had significantly higher *E. coli* counts. For other types of contaminations, the probability of increasing the total bacterial load by at least 0.5 log_10_ was close to 0%. It is important to note that the increase by 0.5 log_10_CFU is approximately equal to a three-fold increase in bacterial load calculated based on CFUx10^x^ values. It might be useful to compare bacterial loads expressed in different units. In our study, the probability of total bacterial increase (*E. coli*, carcasses contaminated with feces) by at least 0.5 log_10_ was 16.7%, while the probability of occurrence of at a least three-fold increase was 17.9% (Figure 3).

These calculations confirmed that the majority of small contaminations have a negligible impact. Although the numbers of bacteria can be substantial on the spot where the small contamination has taken place (especially if feces are involved), when these numbers are related to the total bacterial load that is already present on a whole carcass, the impact of the small contamination becomes negligible (Table 6), consequently causing no extra threat to food safety. This is illustrated by a study by Giombelli and Gloria [21], who found that visible fecal contamination did not influence the counts of *Campylobacter* spp. on the carcasses *per se*, but that it did result in a higher prevalence of *Campylobacter*-positive carcasses, i.e., the number of positive carcasses was higher in the group of fecally contaminated carcasses than in the group of carcasses without any visible fecal contamination. This was also the case in a laboratory experiment on carcass contamination with 0.1 g of feces with cultured bacteria [42]. In addition, when the effects of cooling are taken into consideration, the effects will even be further diminished. As demonstrated by Cibin et al. [18], the cooling process reduced the overall bacterial levels to such an extent that, even in situations where there are significant differences between clean and visibly soiled carcasses at the end of slaughter, the cooling process renders these differences insignificant. Similar results were observed by Cason et al. [43], who reported no differences in bacterial counts post-chilling between carcass halves, from which one was not contaminated, while the other was artificially contaminated with fresh feces.

Many cases of visibly contaminated carcasses can be attributed to a faulty evisceration process. Machines can only be adjusted to work within a certain set of size ranges. Therefore, it would be highly desirable if the machines could be auto-adjusted in real time to the size of every single carcass processed, thus minimizing the risk of faulty evisceration (e.g., intestine or gall bladder disruption) and decreasing the prevalence of visibly contaminated carcasses. However, from the slaughterhouse’s perspective, the reason for the carcass damage can be explained as the lack of uniformity of the delivered broiler flock. In other words, the birds do not meet the expected standard size, which should be the responsibility of the poultry producer. Maintenance of the equipment (or not appropriate maintenance) could also result in poultry carcass damage, including rupture of the gastrointestinal tract. Nevertheless, there are some interventions that aim at reducing bacterial counts on chicken carcasses. For example, the application of rapid surface cooling (immersion in liquid nitrogen) resulted in a reduction of counts of *Campylobacter* spp. by 1 log_10_ CFU/g on the chicken carcass skin [44]. Similar promising results were obtained when the combination of steam and ultrasound were used in the evisceration room, before the inside/outside carcass washer [45]. Carcass trimming or using water sprays to remove contaminations, however, offer no real solution. For example, a study by Giombelli and Gloria [21] showed that there were no differences in bacterial counts before and after the trimming of carcasses with visible fecal contamination, while the water spray (potable water) decreased bacterial loads by approx. 20% (i.e., a factor of 0.8, which in practice will not yield significant impact). Stefani et al. [46] also showed that the washing of carcasses with fecal contamination is more effective in reducing bacterial loads than trimming is. It is not surprising that, in practice, despite all of the actions taken, some carcasses will remain visibly contaminated until just before cooling. However, when seen in the light of our calculations regarding small contaminations, a zero-tolerance policy towards all visible contaminations by some food safety authorities in Europe can be seen as a mainly quality or politically driven and not a real food safety issue. As amply demonstrated by the results of Cibin et al. [18], high microbial counts of carcasses can also occur without visible contamination.

Inspecting chicken carcasses for small visible contaminations by the competent authorities is time-consuming, requires sharp eyesight, and can be highly subjective. Hence, more attention should be paid to robust hygiene criteria, which are far more effective than implementing a strict zero-tolerance policy towards small visible contaminations by the competent authorities. For example, the current process hygiene criterium for poultry production is a *Campylobacter* spp. count with an upper limit set at 1000 CFU/g [47]. For every batch, no more than 15 samples out of 50 should exceed this limit, but the aim is to reduce this number to 10 by 2025. As time progresses, adopting more stringent microbial criteria appears reasonable because producers have ample time to adjust and can at the same time claim to work actively with the competent authorities towards reducing the prevalence of food-borne diseases in humans. For example, a more stringent critical limit for *Campylobacter* spp. of 100 CFU/g could reduce consumer risk of campylobacteriosis via poultry by 98%, but currently, over 55% or more of the batches would not comply [48]; thus, this is currently an impossible criterium to adopt. Producers should still check for small contaminations since they might be the indicators that mechanical adjustments are required during the processing. While the meat inspectors should be also aware of this issue, they should at the same time try to focus on other indicators with well-established food safety implications, as stated above.

## 5. Conclusions

In conclusion, our calculations revealed that carcass contamination with minute amounts of feces, bile, grain from the crop, and grease from the lines will not lead to a significant increase of the already present food safety hazards. Maintaining a strict zero tolerance for these small contaminations on chicken carcasses does not improve the level of protection of the consumer. Instead, it would be far more effective to pay more attention to existing hygienic microbial criteria and a further improvement by a gradual tightening up of these regulations in the future. However, it is important to note that the biological hazards discussed above are best controlled at earlier stages of the production. Ensuring the highest possible animal health status as well as animal welfare standards should play a key role in minimizing food-borne risks for consumers.

## Figures and Tables

**Table 1:** Assumptions regarding small contaminations on broiler carcasses as input for the Monte Carlo simulation.

Type of Contamination	Minimal Amount (g)	Maximal Amount (g)
Feces	0.001	0.01
Bile	0.0375	0.15
Crop content	0.05	0.2
Grease	0.01	0.04

**Table 2:** Number of bacteria per gram in different types of contaminations expressed in log_10_.

	Total Aerobic Count	*Enterobacteriaceae*	*E. coli*	*Campylobacter* spp.
Skin				
mean ± sd (log_10_/g)	4.15 ± 0.46	3.77 ± 0.13	3.3 ± 0.6	2.99 ± 0.7
reference	[30]	[31]	[32]	[26]
Feces				
mean ± sd (log_10_/g)	3.36 ± 1.37	8.62 ± 0.58	8.44 ± 0.35	6.0 ± 1.52
reference	[33]	[34]	[34]	[26]
Bile				
mean ± sd (log_10_/g)	6.0 ± 0.7	3.1 ± 0.5	1.9 ± 1.1	2.795 ± 1.641
reference	[35]	[36]	[37]	[38]
Crop content				
mean ± sd (log_10_/g)	5.6 ± 0.1	4.2 ± 0.2	3.9 ± 0.2	3.63 ± 1.12
reference	[39]	[39]	[39]	[40]
Grease				
mean ± sd (log_10_/g)	3.40 ± 0.16	1.86 ± 0.41	0.86 ± 1.19	0.83 ± 0.67
reference	own data not published	own data not published	own data not published	own data not published

**Table 3:** Monte Carlo simulation of the total bacterial load on the average broiler carcass (1600 g) with or without a contamination with a small amount of material (expressed in log_10_CFU).

Bacterial Species	Type of Contamination							
	Feces		Bile		Crop Content		Grease	
	No Contam.	With Contam.	No Contam.	With Contam.	No Contam.	With Contam.	No Contam.	With Contam.
Total aerobic count								
mean	7.3535	7.3536	7.3535	7.3625	7.3535	7.3552	7.3535	7.3535
sd	0.4604	0.4604	0.4606	0.4528	0.4603	0.4585	0.4606	0.4605
minimum	5.3536	5.3536	5.3394	5.5386	5.4267	5.4681	5.4143	5.4144
maximum	9.3717	9.3717	9.4324	9.4324	9.4114	9.4114	9.2766	9.2766
*Enterobacteriaceae*								
mean	6.9735	7.1181	6.9735	6.9735	6.9735	6.9736	6.9735	6.9735
sd	0.1316	0.1907	0.1318	0.1318	0.1319	0.1319	0.1320	0.1320
minimum	6.4232	6.4665	6.3800	6.3800	6.4164	6.4171	6.3775	6.3775
maximum	7.5920	8.0262	7.5122	7.5122	7.5480	7.5480	7.5612	7.5612
*E. coli*								
mean	6.5035	6.7640	6.5035	6.5036	6.5035	6.5039	6.5035	6.5035
sd	0.6003	0.4368	0.6003	0.6002	0.6005	0.6000	0.6004	0.6004
minimum	3.5649	5.1184	3.7284	3.7287	3.9146	4.0222	3.9726	3.9726
maximum	9.1898	9.1915	9.15620	9.1562	9.2393	9.2393	9.0849	9.0849
*Campylobacter* spp.								
mean	6.1936	6.2610	6.1936	6.2011	6.1936	6.2005	6.1936	6.1936
sd	0.7001	0.6784	0.7002	0.6946	0.7002	0.6931	0.7003	0.7003
minimum	3.3425	3.5869	3.3131	3.3245	3.3419	3.60291	3.2279	3.2287
maximum	9.2117	9.2117	9.26841	9.2684	9.10343	9.10343	9.1931	9.1931

**Table 4:** The probability (%) that a small visible contamination results in at least a 0.5 (log_10_CFU) increase in the total bacterial load of the average chicken carcass (1600 g).

Type of Bacteria	Feces	Bile	Crop Content	Grease
Total aerobic count	0%	0%	0%	0%
*Enterobacteriaceae*	5.1%	0%	0%	0%
*E. coli*	16.7%	0%	0%	0%
*Campylobacter* spp.	4.1%	0%	0.2%	0%

**Table 5:** The probability (%) that a small visible contamination results in at least a three-fold increase (calculated based on CFUx10^x^) in the total bacterial load of the average chicken carcass (1600 g).

Type of Bacteria	Feces	Bile	Crop Content	Grease
Total aerobic count	0%	0%	0%	0%
*Enterobacteriaceae*	5.6%	0%	0%	0%
*E. coli*	17.9%	0%	0%	0%
*Campylobacter* spp.	4.3%	0%	0.2%	0%

**Table 6:** The contribution (%) of small contaminations to the total bacterial load of the average chicken carcass (1600 g).

Type of Bacteria	Feces	Bile	Crop Content	Grease
Total aerobic count	0.001%	0.122%	0.02311%	<0.001%
*Enterobacteriaceae*	2.031%	<0.001%	0.00143%	<0.001%
*E. coli*	3.851%	0.002%	0.00615%	<0.001%
*Campylobacter* spp.	1.077%	0.121%	0.11128%	<0.001%

## Data Availability

Data is contained within the article or supplementary material.

## Supplementary Materials

The following supporting information can be downloaded at: https://www.mdpi.com/article/10.3390/foods12030522/s1, Spreadsheet S1: The baseline model for used for Monte Carlo simulation.
